# High Throughput Screening of Valganciclovir in Acidic Microenvironments of Polyester Thin Films

**DOI:** 10.3390/ma8041714

**Published:** 2015-04-13

**Authors:** Teilo Schaller, Tobias Wenner, Rupesh Agrawal, Stephen Teoh, Li Ting Phua, Joachim S. C. Loo, Terry W. J. Steele

**Affiliations:** 1 School of Materials Science & Engineering, Nanyang Technological University, 50 Nanyang Drive, Singapore 639798, Singapore; E-Mails: teilo.schaller@gmail.com (T.S.); Tobias.Wenner@live.de (T.W.); PHUA0076@e.ntu.edu.sg (L.T.P.); joachimloo@ntu.edu.sg (J.S.C.L.); 2 National Healthcare Group Eye Institute, Tan Tock Seng Hospital, 11 Jalan Tan Tock Seng, Singapore 308433, Singapore; E-Mails: rupesh_agrawal@ttsh.com.sg (R.A.); teoh_st@yahoo.com.sg (S.T.)

**Keywords:** poly(lactic-co-glycolic acid) (PLGA), polymeric biomaterials, ophthalmic drug delivery, high-throughput, fluorescence spectroscopy, drug-excipient interaction

## Abstract

Ganciclovir and valganciclor are antiviral agents used for the treatment of cytomegalovirus retinitis. The conventional method for administering ganciclovir in cytomegalovirus retinitis patients is repeated intravitreal injections. In order to obviate the possible detrimental effects of repeated intraocular injections, to improve compliance and to eliminate systemic side-effects, we investigated the tuning of the ganciclovir pro-drug valganciclovir and the release from thin films of poly(lactic-co-glycolic acid) (PLGA), polycaprolactone (PCL), or mixtures of both, as a step towards prototyping periocular valganciclovir implants. To investigate the drug release, we established and evaluated a high throughput fluorescence-based quantification screening assay for the detection of valganciclovir. Our protocol allows quantifying as little as 20 ng of valganciclovir in 96-well polypropylene plates and a 50× faster analysis compared to traditional HPLC measurements. This improvement can hence be extrapolated to other polyester matrix thin film formulations using a high-throughput approach. The acidic microenvironment within the polyester matrix was found to protect valganciclovir from degradation with resultant increases in the half-life of the drug in the periocular implant to 100 days. Linear release profiles were obtained using the pure polyester polymers for 10 days and 60 days formulations; however, gross phase separations of PCL and acid-terminated PLGA prevented tuning within these timeframes due to the phase separation of the polymer, valganciclovir, or both.

## 1. Introduction

The cytomegalovirus (CMV) is a member of the herpesviridae family and infects approximately 50% of the European population [1,2]. As a herpes virus, CMV is characterized by lifelong persistence in the host [3,4]. Chronic infection of the host usually remains subclinical, as the virus count is actively kept under control by the immune system. Nonetheless, in immunocompromised patients, such as HIV patients, latent cytomegalovirus may reactivate and become life threatening. Cytomegalovirus retinitis (CMVR) is the most common opportunistic AIDS-related ocular complication and occurs in 40% of all HIV patients with CD4 counts of less than 50 units [5].

Ganciclovir, an inhibitor of the viral DNA polymerase is the conventional drug to treat CMVR [6]. However, due to the poor oral bioavailability, it requires intravenous injections, intravitreal injections, or ocular implants [7]. High therapeutic serum concentration is needed for the parenteral treatment of CMVR requiring hospitalization with an increased risk for adverse systemic reactions [8]. In addition to the induction regimen (intravitreal injections of ganciclovir twice a week for one month), a further maintenance phase is required (weekly injection) until resolution of the lesion and reconstitution of the immune system, eventually requiring patients to undergo a minimum of 16 intravitreal injections over a period of three months. Repeated intravitreal injections are prone to cause detrimental effects in the eye including, but not limited to, retinal detachment, cataract, endophthalmitis, vitreous hemorrhage, and blindness [1,9]. The advent of valganciclovir, an L-valyl ester prodrug of ganciclovir, shows marked improvement in bioavailability of ganciclovir due to its transport via the peptide transporter 1 (PEPT1), hence allowing peroral intake of the drug [10,11]. However, due to its prohibitive cost and dubious efficacy for vision threatening CMVR, the popularity of this regimen is still very limited amongst physicians. Nonetheless, to obviate the systemic administration and frequent repeated intravitreal injections, scientists have shifted their focus on new methods for localized drug delivery.

The spectrum of ocular drug delivery systems cover a variety of classes, including biodegradable and non-biodegradable intravitreal implants, emulsions, microspheres, and polymer formulations [12]. The main advantage of drug delivery systems is a controlled drug release near the target area which results in stable drug concentrations. This reduces the risk of toxicity caused by peak concentrations and lowers the risk of resistance due to constant drug levels. Furthermore, drug delivery systems result in improved patient compliance due to long-term release formulas that eliminate treatments consisting of undesirable injections or the need for a daily pill regimen.

Polyesters, particularly poly(lactic-co-glycolic acid) (PLGA) and polycaprolactone (PCL), have been incorporated into numerous biodegradable drug delivery systems due to their proven biocompatibility and bioresorption properties [12].

The proposed development of a prototype biodegradable thin film valganciclovir-eluting disc designed for periocular implantation may facilitate long-term CMVR treatment, obviating the need for systemic or intravitreal therapy for CMVR. The major benefits speculated for such a prototype would be sustained release drug levels, higher compliance, cost-effective implantation, and the avoidance of complications from intravitreal injections. The rationale for using valganciclovir instead of ganciclovir for the proposed therapy is increased membrane permeability of valganciclovir due to its lesser hydrophilic character and its transport via PEPT1 expressed in the eye [13].

Our laboratory has previously published on both high-throughput drug release measurements and on tuning the drug release of fluorescein diacetate in polyester thin film using acidic terminal end-groups to control the rate of degradation [14,15,16]. However, the measurement of valganciclovir in drug release studies has only been conducted with time-consuming analytical methods such as HPLC.

High-throughput fluorescent screening of valganciclovir drug release and the ability to tune that drug release is explored in this research project for the first time. Furthermore, there are no reports of factors affecting valganciclovir stability inside polyester mixtures, an important prerequisite which will be investigated in this study. We hypothesize that the drug release of valganciclovir can be tuned by varying the acidity of homogenous polyester thin films using differing ratios of the acid-terminated PLGA and PCL, which can best be assessed with a high-throughput quantitation method.

## 2. Materials and Methods

### 2.1. Materials

Poly (DL-lactide-co-glycolide) 53/47 (lactide-glycolide ratio) (PLGA) #PDLG 5002A with inherent viscosity 0.2 dL/g was purchased from Purac, Gorinchem, Netherlands; valganciclovir from Afine Chemicals Ltd., Hangzhou, China; and ganciclovir from Sinoright, Dalian, China.

### 2.2. Preparation and Casting of PLGA/PCL Thin Films

A total of 800 mg of one or both polymers was weighed and dissolved in dichloromethane. In addition to dissolving the polymers alone, ratios of 10:1, 8:1, 6:1, 4:1, 2:1, 1:1, 1:4, and 1:10 poly(lactic-co-glycolic acid) (PLGA) to polycaprolactone (PCL) (w:w) mixtures were prepared. 5%, 10%, and 20% (w/w) valganciclovir were dissolved in methanol and added to each polymer solution. The solutions were cast on polyethylene terephthalate (PET) using the 1137 Automatic Film Applicator from Paul N. Gardner Company, Pompano Beach, FL, USA. Punchouts of the films were made using a stancing tool.

### 2.3. Spectrofluorometer Measurements of Valganciclovir

Valganciclovir punchouts were incubated in 200 µL of PBS media. Samples were incubated at 37 °C in an oven, with media change and fluorescence measurements occurring at baseline (0 h), 1 h, and 6 h, every day until day seven, every 2 days until day 21, and every 4 days until 60 days. Remaining valganciclovir left inside the films after 60 days was measured by dissolving the films in dimethylformamide (DMF). Spectrofluorometer measurements were done by pipetting 150 µL of release medium (PBS buffer at 7.4 pH) into polypropylene wells and subsequently adding 6 µL of 18.5% hydrochloric acid (HCl). Fluorescence of valganciclovir in Greiner 96-well black polypropylene plates from Sigma-Aldrich, Singapore was measured using an Infinite 200 Pro Plate Fluorescence Reader from Tecan Group Ltd., San Jose, CA, USA.

### 2.4. Polymer, Valganciclovir, and Thickness Quantification by 1H-NMR

Three dried punchouts of each film were dissolved in 700 µL of deuterated DMF and vortexed before transferring them to NMR tubes. 1H-NMR spectra were recorded on a Bruker Advance Spectrometer at 400 MHz using 0.05% Benzene as an internal standard. 1H-NMR (400 MHZ, d-DMF, δ) 0.7–0.8 [s, valganciclovir 6H, -C(CH3)2], 2.05–2.2 [t, PCL 2H], 2.65–2.75 [s, dDMF], 4.75–4.85 [m, PLGA 2H, -C(=O)-(CH3)-O-], 5.0–5.1 [m, PLGA 1H, -C(=O)-CH(CH3)-O-], 7.1–7.2 [s, Benzene]. The NMR error was calculated using the combined error from d6-DMF peak deviations (weigh in error) and the standard deviation of the lactide/glycolide ratios (integration and machine error). Film thickness was estimated from the calculated punchout density using the known densities of PLGA, PCL, and valganciclovir combined with the NMR composition data. The punchout density (in µg/cm^3^) was divided by the NMR generated mass of PLGA, PCL, and valganciclovir per punchout (in µg/cm^2^), giving the average film thickness. The method has been described previously [15,17].

### 2.5. Valganciclovir Conversion to Ganciclovir Inside of Thin Film by HPLC Quantification

Punchouts with a 1:1 polymer ratio from 10% and 20% valganciclovir films were incubated in drug release study conditions at 37 °C in 200 µL of PBS. PBS was replaced daily. After every 24 h, three punchouts from each of the two films were removed, rinsed with PBS, dried under a vacuum, and dissolved in 0.5 mL of chloroform. Next, 1 mL of PBS was added, and the mixture was shaken and sonicated for 15 min. After separation of the phases, the valganciclovir and/or ganciclovir containing solution was removed and filtered with a 0.22 syringe filter. For HPLC analysis, 40 µL were injected into the Agilent 1100 chromatographic system using a Poroshell 120 EC-C18 column and a spectrofluorometer (Model 1260). Valganciclovir and ganciclovir were separated isocratically with a flow rate of 0.9 mL/min, using 3.2% acetonitrile in 0.05 M dihydrogen phosphate buffer with added phosphoric acid at 2.5 pH. Measurements were done at λ(EX) = 265 nm and λ(EM) = 375 nm. Half-life determination was assessed by measuring the fluorescence of valganciclovir (F) over time (t) allows calculating the degradation constant (k) according to the equation A(t) = A(0) exp(−kt). Half-life (t_1/2_) was determined by the equation t_1/2_ = ln(2)/k.

### 2.6. Statistics Method Analysis

Linear regressions and Pearson’s correlations were calculated with Origin 8.5 SRI. Pearson’s correlations were determined with a minimum of *n* = 5 data points.

## 3. Results

### 3.1. High-Throughput Screening Leads to 50x Faster Development

**Figure 1:**
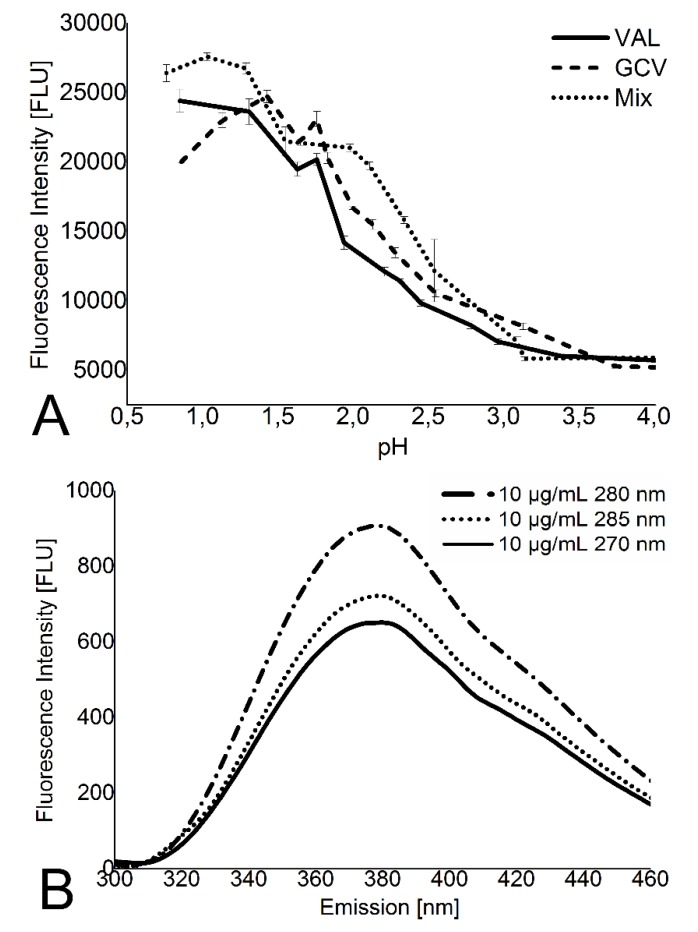
Optimization for parameters for fluorescence measurement. (**A**) Equimolar concentrations of valganciclovir (VAL) and ganciclovir (GCV) as well as a 1:1 mixture (Mix) were tested for pH-dependent fluorescence change. Valganciclovir and ganciclovir seem to overlap at a pH value close to 1.2 and close to 3.6. The overlap close to pH 1 has the higher fluorescence intensity of the two pH values; (**B**) Enlarged emission spectrum of valganciclovir at 10 µg/mL showing the global maximum for different excitation wavelength.

To develop a high-throughput screening for valganciclovir detection, buffer conditions were optimized using a quartz cuvette. Optimization screening found a pH-dependent fluorescence increase in acid conditions with a maximum fluorescence below pH 1. pH 1 was used for all subsequent fluorescence measurements (Figure 1A). The excitation maximum was 280 nm at valganciclovir concentrations at or below 10 µg/mL (Figure 1B; data points only shown for 10 µg/mL).

Different 96-well plate materials were tested to minimize autofluorescence. polystyrene showed an autofluorescence signal of 302 ± 8.2 fluorescence units (FLU) at the measurement wavelength, which is 1.7 times higher than the noise fluctuations of the detector. Autofluorescence of the polypropylene and quartz glass plates at 175 ± 3.3 and 183 ± 3.8 was minimal and showed no significant difference when compared to the fluorescence noise of the detector. All subsequent fluorescence measurements were therefore done with disposable 96-well polypropylene plates.

Calibration curves from 100 ng/mL to 100 µg/mL valganciclovir were made. A limit of quantitation (LOQ) of 100 ng/mL was calculated for valganciclovir using the polypropylene plates in the Tecan plate reader.

Previous investigations have shown that the phenylglyoxal can fluorescently label guanine-like structures, such as ganciclovir and valganciclovir [18,19]. However, the LOQ in the Tecan Plate reader showed no improvement over valganciclovir’s intrinsic fluorescence (data not shown), and the required additional labor and reagents hindered the high throughput nature of the bioassay. Therefore, phenylglyoxal labeling wasn’t utilized in our investigations. Nevertheless, one advantage of the phenylglyoxal labeling is a shift in excitation to a lower energy wavelength (365 *vs.* 280 nm), which allows for detection in a broader spectrum of release mediums such as those that may contain proteins or other nucleic acids, e.g., human blood plasma.

Half-life determination of valganciclovir showed that the degradation followed first order kinetics. The half-life in PBS with pH 7.4 at T = 23.5 °C was calculated at t_1/2_ = 36.7 h ± 0.3. At T = 37 °C, a t_1/2_ = 10.7 ± 0.3 h was measured, which agrees with the 11 h half-life determined by Li *et al.* in similar conditions [20]. At pH 1 and T = 23.5 °C, valganciclovir showed virtually no degradation over a time period of 22 h, signifying that the drug is stable for several hours before quantitation needs to be done—an important aspect for automated high-throughput screening protocols.

The NMR measurements of four thin film formulations, listed in Table 1, revealed that the PLGA:PCL ratios, valganciclovir content, and thickness measurements matched the predicted results. Film thickness measurements using NMR are more time efficient compared to other methods, such as microscopy [15,17].

**Table 1:** NMR quantification of the valganciclovir polymer films.

Thin Film	Valganciclovir (µg/cm^2^)	Valganciclovir added (%)	PLGA (µg/cm^2^)	PCL (µg/cm^2^)	PLGA:PCL	NMR estimated * thickness (µm)
PLGA	106 ± 4	2.8 ± 0.1	3900 ± 130	0.0 ± 0.0	N/A	29.4 ± 1.0
1:4 PLGA: PCL	805 ± 27	9.9 ± 0.3	1540 ± 50	7260 ± 250	1:4.71	79.1 ± 2.7
1:10 PLGA: PCL	352 ± 12	7.1 ± 0.2	506 ± 17	4800 ± 160	1:9.54	47.8 ± 1.6
PCL	411 ± 14	12.6 ± 0.4	196 ± 7	3500 ± 120	N/A	34.2 ± 1.2

* based on density and mass per cm^2^.

The time required for the high throughput fluorescence assay, including setup of the machine, sample preparation, and running samples, is less than 20 min per 96-well plate. Running 96 samples using the standard HPLC based detection assay requires approximately 16 h. When using the high throughput fluorescence assay we developed, there is an approximate 50-fold increase in throughput as well as significant savings in consumables (no HPLC solvents or columns).

### 3.2. Valganciclovir Proves to be Stable within Polyester Films

**Figure 2:**
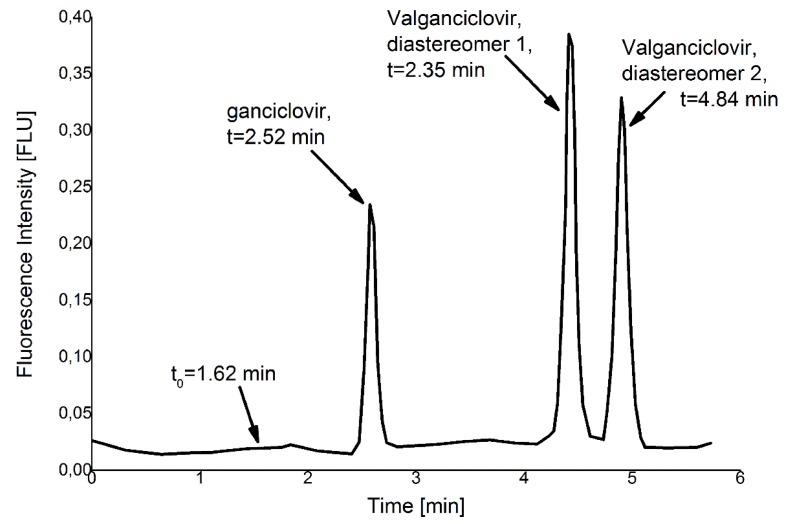
Replotted HPLC chromatogram of valganciclovir normalized with blank. Inj. Volume: 40 µL; ganciclovir is eluted after t(GCV) = 2.52 ± 0.01 min and the two peaks representing the diastereomers of valganciclovir eluted at t(VAL1) = 4.35 ± 0.06 min and t(VAL2) = 4.84 ± 0.06 min. All peaks are separated and allow quantitation.

HPLC analysis showed that there was minimal degradation of valganciclovir to ganciclovir in the films containing 1:1 PLGA to PCL inside of the thin film after 7 days of incubation at 37 °C. Peaks for the two valganciclovir diastereomers and the ganciclovir as well as the retention times can be seen in Figure 2. Day 0 punchouts of the 20% valganciclovir films showed a 99.5% ± 0.3% valganciclovir to 0.5% ± 0.3% ganciclovir ratio (mol/mol). After 7 days, the valganciclovir proportion dropped to 96.8% + 0.1%. For the 10% valganciclovir films, the unincubated punchouts after 7 days showed a 100.1% ± 0.1% valganciclovir proportion and the incubated punchouts showed a 95.9% ± 0.8% valganciclovir proportion. The half-life for valganciclovir in the 20% drug polyester film was calculated to be 95.0 days (81.5 days to 113.6 days) and 118.5 days (109.5 days to 129.1 days) for the 10% valganciclovir film. Comparing these two half-lives to the half-lives of valganciclovir reported by Li *et al.* [20] 1999 at different pH, suggests an environmental pH of approximately 4.4 ± 0.3. Although there are numerous reports on the pH microclimate of PLGA with varying pH values, they all agree on an increasingly acidic pH ranging from 1.5 to 4.7 [21,22,23]. Comparing the half-life of valganciclovir in PBS at pH 7.4, measured to be 10.6 h (10.2 to 11.1 h), to in-film degradation shows a dramatic increase in stability.

### 3.3. Pure Films Allow Short and Long Term Valganciclovir Drug Delivery, but Polymer Phase Separation Prevents Tuning of Drug Release

**Figure 3:**
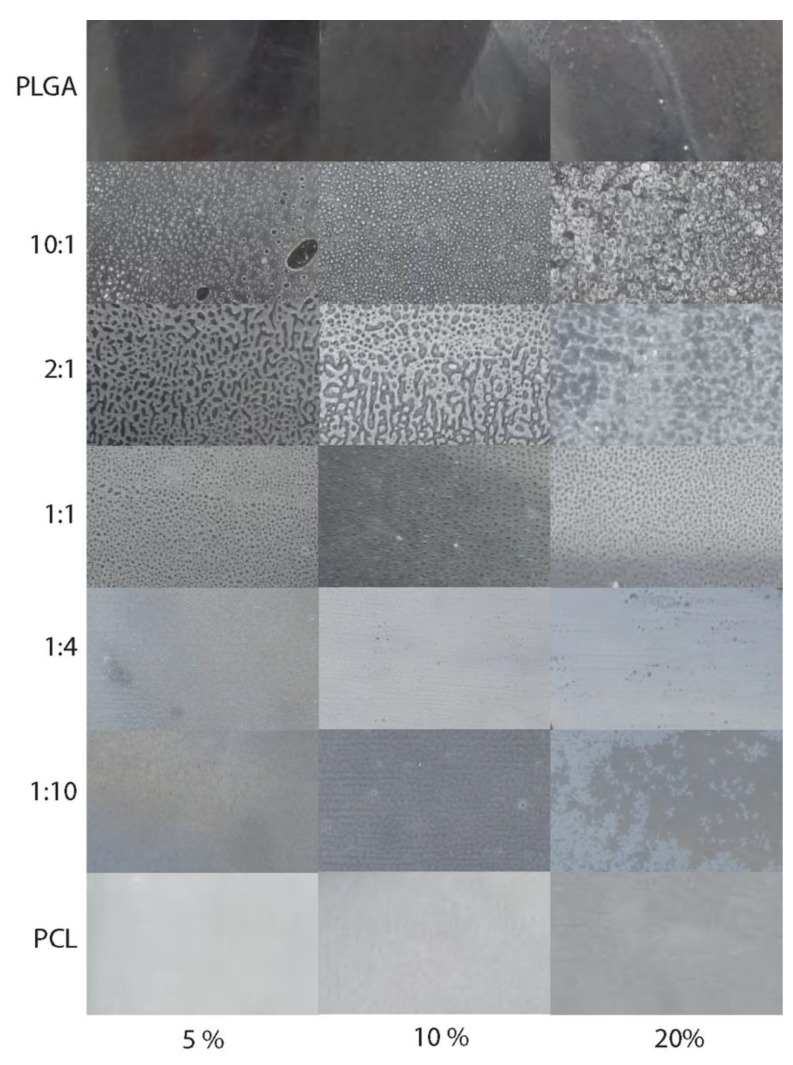
Surface of the thin films. Matrix showing the casted thin films with differing ratios of PLGA to PCL. From top to bottom the PCL content in the films increases, from left to right the valganciclovir content increases from 5% over 10% to 20% drug added. The following polymer ratios are shown: Pure PLGA (row 1), PLGA:PCL = 10:1 (row 2), 2:1 (row 3), 1:1 (row 4), 1:4 (row 5), 1:10 (row 6) and pure PCL (row 7). A phase separation seems to have occurred in the films with more PLGA than PCL and in the films with a 1:1 ratio.

Casting of the thin films revealed phase separations in all of the mixed polymer films, which increased with higher PLGA to PCL ratios (Figure 3). Tuning of the long-term drug release study for these polymer mixtures was effectively prevented by this phase separation.

**Figure 4:**
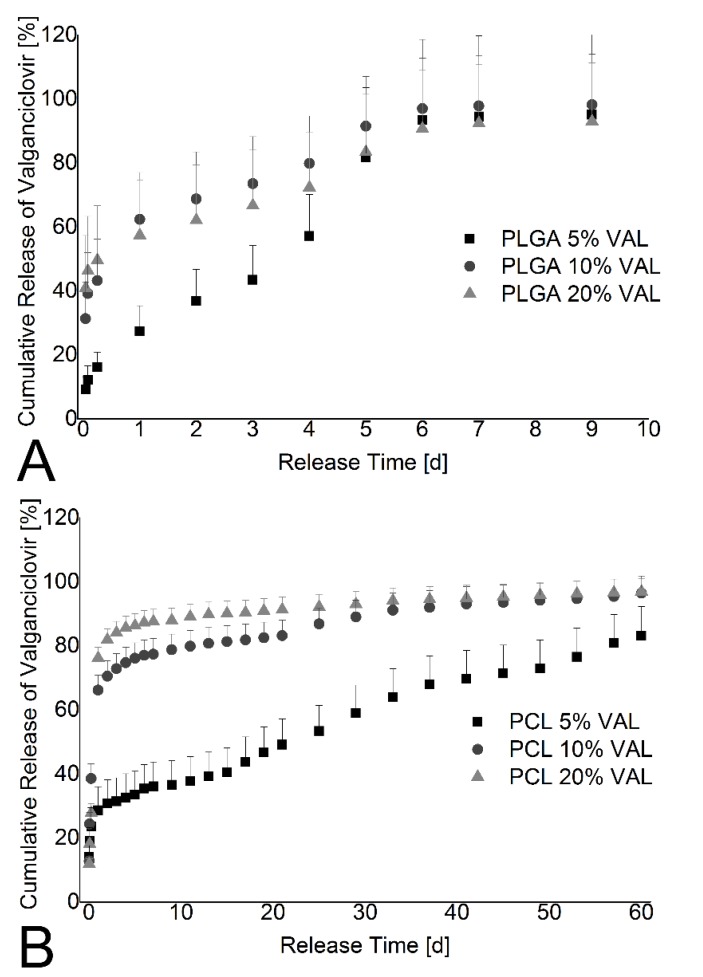
Cumulative release of valganciclovir in percent. The release is plotted in percent of total drug amount of each punchout *versus* the incubation time. The rectangles are the 5% valganciclovir films, the circle the 10% valganciclovir films, and the triangle the 20% valganciclovir films. Both PLGA and PCL films show significant burst releases in the first day. (**A**) Drug release profile of PLGA thin film over a time period of 10 days showing that drug release can be controlled, but with limited dependence on valganciclovir content. After six days, all three thin films have completely released their valganciclovir content; (**B**) Drug release profile of PCL thin film with a controlled release over a time period of 60 days showing a burst release dependent on the amount of valganciclovir added. The 5% pure PCL film shows a lower than usual amount of drug release (<50 µg/cm^2^); however, a clear drug release profile can be seen.

Nevertheless, the pure PLGA and PCL films allow short and long term valganciclovir drug delivery, respectively (Figure 4). Increasing the initial amount of valganciclovir in the PCL film increases the burst release, which was not observed for the PLGA film (Figure 4).

**Figure 5:**
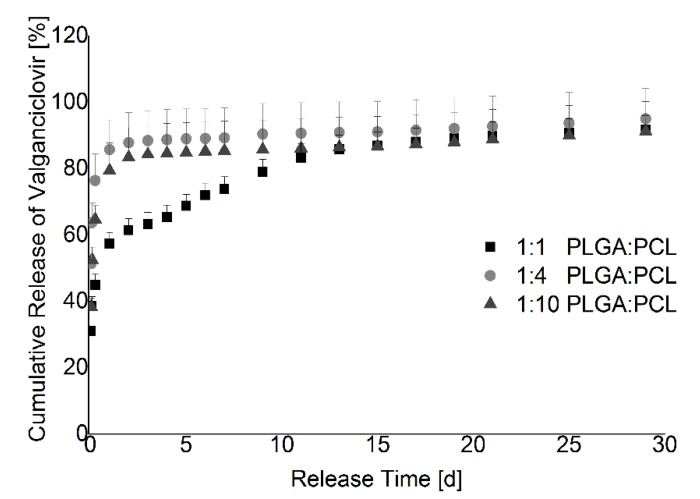
Cumulative release of valganciclovir out of films with more PCL. The graph shows the percentage of released drug out of 5% valganciclovir films *versus* the release time. Data for the film with PLGA and PCL in equal mass concentrations are depicted by the rectangles, the 1:4 PLGA to PCL film by the circles, and the 1:10 PLGA to PCL film by the triangles. All other polymer ratio films showed phase separation and were not evaluated. Nevertheless, the plotted films show a high burst release, even without visible phase separation.

Figure 5 shows the drug release of 5% valganciclovir thin films with 1:1, 1:4, and 1:10 PLGA to PCL ratios. These are the films with the least amount of visible phase separation. Nevertheless, over a time period of 30 d, little to no drug release tuning could be realized. Should phase separation be improved or eliminated in further studies, the thickness differences seen in the thin films, as shown by ^1^H NMR measurements in Table 1, may still interfere with comparing drug release from thin films with different ratios of PLGA to PCL.

## 4. Discussion

### 4.1. High-Throughput Screening Shows Significant Improvement Compared to HPLC

The benefits of high-throughput methods are obvious: more analysis in a shorter time results in more time for research experiments. In most cases, such as in this study, high-throughput also increases cost-effectiveness.

The establishment of a high-throughput valganciclovir detection method in a plate reader based upon the drug’s intrinsic fluorescence is described in this paper for the first time. The 50× rate increase of sample throughput, when compared to the standard valganciclovir detection method in the HPLC, is substantial and changes the rate limiting factor from the machine to the scientist.

Using a fluorescence plate reader allows for the measurement of one 96 sample set in less than 20 min, whereas the HPLC requires around 16 h. This efficiency increase makes it possible to increase the measurement interval of the drug release study and thereby allows for more exact drug release curves, or for an increase in the number of sample sets. Hypothetically, a scientist working 8 h/d would be able to prepare and measure more than 2304 drug release samples (or 24 plates/d). In addition to the economic benefits that arise from the substantial speed increase, use of the plate reader also provides environmental benefits in comparison to the HPLC. At a flow rate of 0.9 mL/min, the HPLC consumes 9.8 L of buffer per sample set, requires 96 one-time-use HPLC vials and micro vial insets, and degrades the column for 14.7 h—All of which can be avoided by using the fluorescence plate reader.

The sensitivity of the method was optimized by adjusting the pH, tuning excitation and emission wavelengths, and minimizing the autofluorescence of the 96-well plates. Our study demonstrates that the fluorescence of valganciclovir and ganciclovir is strongly pH-dependent and the fluorescence intensity improves with low pH. These results correlate with the findings of Mascher *et al.* [24] which demonstrated the same for aciclovir, another guanine-like drug. The long stability of valganciclovir in acidic conditions is a benefit for this detection method because degradation will occur neither during analysis nor during storage of the valganciclovir solutions [2]. Polypropylene plates are ideal for the detection of valganciclovir as they show negligible autofluorescence, are sterile, disposable, compatible with most solvents (unlike polystyrene), and cost-efficient.

The 100 ng/mL limit of quantification for valganciclovir that we determined for our high-throughput fluorescence measuring system is one order of magnitude higher than the traditional HPLC [25]. Given the typical valganciclovir concentrations generated by drug eluting matrices, the loss in sensitivity is irrelevant for assessment of *in vitro* release kinetics, but may not be sensitive enough for tissue distribution analyses. Towards *in vitro* release assessment, the loss of sensitivity compared to HPLC is more than compensated by the increase in throughput.

### 4.2. Acidic Microenvironment of Polyester Films Protects Valganciclovir from Physiological pH Degradation

Drugs can be released out of polymer matrices by different mechanisms. A common method is to modify the drug release by adding specific substances called additives or excipients. These components have to be biocompatible.

We demonstrate that a polymer matrix, consisting of two different biocompatible polymers, has an influence on the drug behavior. By varying the ratio of the two components PLGA and PCL, one varies the acidity of the PLGA:PCL film. The acidity can influence the degradation rate of the polymers as well as the degradation rate of valganciclovir [2]. We show that the half-life of valganciclovir is prolonged from 10.6 hours in PBS buffer to approximately 100 days inside of 1:1 PLGA to PCL thin films.

The microenvironment of PLGA is acidic in nature [23]. With a direct link between the half-life of valganciclovir to pH, we suggest that the intrinsic low pH of the polymer mixture provides an acidic microenvironment that inhibits valganciclovir from degrading during our drug release trials [20]. It is this increase in half-life inside of the polymer that makes it feasible to consider PLGA thin films as well-suited drug reservoirs for valganciclovir.

The drawbacks of other ophthalmic drug delivery systems, such as microemulsions, microspheres, and liposomes, indicate that the development of a valganciclovir release system is challenging [26,27,28]. Our approach shows that using PLGA and PCL thin films provides a biodegradable and stable release platform for valganciclovir drug release. We speculate that by implanting the valganciclovir-infused thin films into sub-tenon’s or periocular space, it would be possible to prevent rapid valganciclovir degradation and allow transport into the intravitreal space via the PEPT1 peptide transporter as well as transport by diffusion [11].

### 4.3. Pure Films Allow Short and Long Term Valganciclovir Drug Delivery, but Polymer Phase Separation Prevents Tuning of Drug Release

The phase separation between acid-terminated PLGA and PCL in the thin films effectively prevented the tuning of the drug release due to high burst release of valganciclovir from the thin films. The burst release is caused by the phase separation of PLGA, PCL, and valganciclovir as it allows for rapid diffusion of PBS into the thin film, subsequently allowing valganciclovir to rapidly dissolve into the surrounding medium. Interestingly, phase separation is most visible in films with high PLGA to PCL ratios. As the polymer ratio shifts toward PCL, the phase separation appeared to decrease (Figure 3). The opacity of the PCL-containing thin films may also be an indication that valganciclovir is incorporated in a crystalline form, leading to a high burst release as seen in Figure 4B. It is apparent that the burst release increases with higher valganciclovir concentrations. However, after accounting for drug lost due to the burst release, the pure PCL films, despite differing initial concentrations of valganciclovir, contain a similar concentration of drug. This leads to the hypothesis that we have reached the solubility limit of valganciclovir in PCL. While burst release is normally hard to control, it should be evaluated as to whether or not a small burst release can be used as a strategy to shorten the initial time needed to reach therapeutic concentrations. *In vitro* drug release studies using scleral tissues are needed in order to test whether the burst release in combination with the subsequent drug release from the thin films allow reaching the minimum inhibitory concentration needed for the treatment of CMVR.

In contrast to the other thin films, the pure PLGA thin films seem to show no phase separation and allows for short term controlled drug release over 10 days (Figure 4A).

We speculate that the three compounds must be completely miscible for tuning and that their properties are too different to be miscible and homogeneous when dried [29,30]. The leakage of hydrophilic drugs from polymer drug delivery systems is known from nanoparticle formulations [31]. One possible alternative is to vary the PGA to PLA ratio of PLGA, as a higher PLA content increases the hydrophobicity and a higher PGA content increases the hydrophilicity [32,33]. The number of free carboxyl groups can increase the incorporation rate of proteins or amino acids, suggesting a higher interaction with valganciclovir [33]. In addition, by changing the polymers we may also be able to improve the miscibility of valganciclovir.

## 5. Conclusions

By optimizing medium pH and by decreasing microplate autofluorescence, we developed an analytical method that provides high-throughput screening of ganciclovir-related adducts. The ability to measure the drug release of valganciclovir from polyester thin films using 96-well plate fluorescence measurements represents a dramatic increase in throughput when compared to standard HPLC protocols. We calculate a 50-fold increase in measurement efficiency, which significantly raises the productivity in *in vitro* drug release experiments, while minimizing consumables and solvents.

The high stability of valganciclovir in low pH makes the acidic microenvironment of the polyester films an ideal drug delivery matrix for short to long-term medical implants. Furthermore, the prolonged half-life of 100 days would allow valganciclovir to retain its superior bioavailability properties, in comparison to ganciclovir, towards ocular treatment of cytomegalovirus retinitis.

## References

[1] Tseng A., Foisy M. (1996). The role of ganciclovir for the management of cytomegalovirus retinitis in HIV patients: Pharmacological review and update on new developments. Can. J. Infect. Dis..

[2] Stefanidis D., Brandl M. (2005). Reactivity of valganciclovir in aqueous solution. Drug Dev. Ind. Pharm..

[3] Alcami A., Koszinowski U.H. (2000). Viral mechanisms of immune evasion. Immunol. Today.

[4] Morlet N., Young S., Naidoo D., Graham G., Coroneo M.T. (1996). High dose intravitreal ganciclovir injection provides a prolonged therapeutic intraocular concentration. Br. J. Ophthalmol..

[5] Jabs D.A. (1995). Ocular manifestations of HIV infection. Trans. Am. Ophthalmol. Soc..

[6] Moss P., Rickinson A. (2005). Cellular immunotherapy for viral infection after HSC transplantation. Nat. Rev. Immunol..

[7] Loregian A., Gatti R., Palu G., de Palo E.F. (2001). Separation methods for acyclovir and related antiviral compounds. J. Chromatogr. B.

[8] Daikos G.L., Pulido J., Kathpalia S.B., Jackson G.G. (1988). Intravenous and intraocular ganciclovir for CMV retinitis in patients with AIDS or chemotherapeutic immunosuppression. Br. J. Ophthalmol..

[9] Stewart J.M., Srivastava S.K., Fung A.E., Mahmoud T.H., Telander D.G., Hariprasad S.M., Ober M.D., Mruthyunjaya P. (2011). Bacterial contamination of needles used for intravitreal injections: A prospective, multicenter study. Ocul. Immunol. Inflamm..

[10] Jung D., Dorr A. (1999). Single-dose pharmacokinetics of valganciclovir in HIV- and CMV-seropositive subjects. J. Clin. Pharmacol..

[11] Sugawara M., Huang W., Fei Y.J., Leibach F.H., Ganapathy V., Ganapathy M.E. (2000). Transport of valganciclovir, a ganciclovir prodrug, via peptide transporters PEPT1 and PEPT2. J. Pharm. Sci..

[12] Choonara Y.E., Pillay V., Danckwerts M.P., Carmichael T.R., du Toit L.C. (2010). A review of implantable intravitreal drug delivery technologies for the treatment of posterior segment eye diseases. J. Pharm. Sci..

[13] Kadam R.S., Vooturi S.K., Kompella U.B. (2013). Immunohistochemical and functional characterization of peptide, organic cation, neutral and basic amino acid, and monocarboxylate drug transporters in human ocular tissues. Drug Metabol. Dispos..

[14] Huang C.L., Kumar S., Tan J.J.Z., Boey F.Y.C., Venkatraman S.S., Steele T.W.J., Loo J.S.C. (2013). Modulating drug release from poly(lactic-co-glycolic acid) thin films through terminal end-groups and molecular weight. Polymer Degrad. Stab..

[15] Steele T.W.J., Huang C.L., Kumar S., Iskandar A., Baoxin A., Boey F.Y.C., Loo J.S.C., Venkatraman S.S. (2013). Tuning drug release in polyester thin films: Terminal end-groups determine specific rates of additive-free controlled drug release. NPG Asia Mater..

[16] Steele T.W.J., Huang C.L., Kumar S., Widjaja E., Boey F.Y.C., Loo J.S., Venkatraman S.S. (2011). High-throughput screening of PLGA thin films utilizing hydrophobic fluorescent dyes for hydrophobic drug compounds. J. Pharm. Sci..

[17] Steele T.W.J., Huang C.L., Kumar S., Irvine S., Boey F.Y.C., Loo J.S., Venkatraman S.S. (2012). Novel gradient casting method provides high-throughput assessment of blended polyester poly(lactic-co-glycolic acid) thin films for parameter optimization. Acta Biomater..

[18] Kai M., Ohkura Y., Yonekura S., Iwasaki M. (1988). Selective determination of guanine and its nucleosides and nucleotides by reaction with phenylglyoxal as a fluorogenic reagent. Anal. Chim. Acta.

[19] Rao M., Prahhaka T., Sankar G., Jyothi N. (2012). Development and validation of new stability indicating HPLC method for the determination of valganciclovir in tablet dosage forms. Int. J. Pharm. Sci..

[20] Li W., Anwar F., Jesurrun J., Erice A. (1999). Cytomegalovirus UL97 and glycoprotein B (gB) sequences in tissues from immunocompromised patients with ganciclovir-resistant virus infection. Scand. J. Infect. Dis..

[21] Fu K., Pack D.W., Klibanov A.M., Langer R. (2000). Visual evidence of acidic environment within degrading poly(lactic-co-glycolic acid) (PLGA) microspheres. Pharm. Res..

[22] Li L., Schwendeman S.P. (2005). Mapping neutral microclimate pH in PLGA microspheres. J. Control. Release.

[23] Mader K., Nitschke S., Stosser R., Borchert H.H., Domb A. (1997). Non-destructive and localized assessment of acidic microenvironments inside biodegradable polyanhydrides by spectral spatial electron paramagnetic resonance imaging. Polymer.

[24] Mascher H., Kikuta C., Metz R., Vergin H. (1992). New, high-sensitivity high-performance liquid-chromatographic method for the determination of acyclovir in human plasma, using fluorometric detection. J. Chromatogr. Biomed. Appl..

[25] Dogan-Topal B., Uslu B., Ozkan S.A. (2007). Development and validation of an RP-HPLC method for determination of valganciclovir in human serum and tablets. Chroma.

[26] Yasukawa T., Tabata Y., Kimura H., Ogura Y. (2011). Recent advances in intraocular drug delivery systems. Recent Pat. Drug Deliv. Formul..

[27] Soppimath K.S., Aminabhavi T.M., Kulkarni A.R., Rudzinski W.E. (2001). Biodegradable polymeric nanoparticles as drug delivery devices. J. Control. Release.

[28] Honda M., Asai T., Oku N., Araki Y., Tanaka M., Ebihara N. (2013). Liposomes and nanotechnology in drug development: Focus on ocular targets. Int. J. Nanomed..

[29] Diban N., Haimi S., Bolhuis-Versteeg L., Teixeira S., Miettinen S., Poot A., Grijpma D., Stamatialis D. (2013). Hollow fibers of poly(lactide-co-glycolide) and poly(epsilon-caprolactone) blends for vascular tissue engineering applications. Acta Biomater..

[30] Lao L.L., Venkatraman S.S., Peppas N.A. (2008). Modeling of drug release from biodegradable polymer blends. Eur. J. Pharm. Biopharm..

[31] Barichello J.M., Morishita M., Takayama K., Nagai T. (1999). Encapsulation of hydrophilic and lipophilic drugs in PLGA nanoparticles by the nanoprecipitation method. Drug Dev. Ind. Pharm..

[32] Panyam J., Williams D., Dash A., Leslie-Pelecky D., Labhasetwar V. (2004). Solid-state solubility influences encapsulation and release of hydrophobic drugs from PLGA/PLA nanoparticles. J. Pharm. Sci..

[33] Hans M.L., Lowman A.M. (2002). Biodegradable nanoparticles for drug delivery and targeting. Curr. Opin. Solid State Mater. Sci..

